# Persistent astrocyte activation in the fragile X mouse cerebellum

**DOI:** 10.1002/brb3.400

**Published:** 2015-09-25

**Authors:** Laura K. K. Pacey, Sihui Guan, Sujeenthar Tharmalingam, Christian Thomsen, David R. Hampson

**Affiliations:** ^1^ Department of Pharmaceutical SciencesLeslie Dan Faculty of PharmacyUniversity of Toronto144 College StreetTorontoOntarioCanadaM5S 3M2; ^2^ Department of NeuroinflammationLundbeck Research USA215 College RoadParamusNew Jersey07652; ^3^ Department of PharmacologyFaculty of MedicineUniversity of Toronto144 College StreetTorontoOntarioCanadaM5S 3M2

**Keywords:** Astrogliosis, autism, FMRP, GFAP, LIF, myelin, oligodendrocyte precursor cells, S100B, TNFR2

## Abstract

**Background:**

Fragile X Syndrome, the most common single gene cause of autism, results from loss of the RNA‐binding protein FMRP. Although FMRP is highly expressed in neurons, it has also recently been identified in glia. It has been postulated that in the absence of FMRP, abnormal function of non‐neuronal cells may contribute to the pathogenesis of the disorder. We previously demonstrated reduced numbers of oligodendrocyte precursor cells and delayed myelination in the cerebellum of fragile X (Fmr1) knockout mice.

**Methods:**

We used quantitative western blotting and immunocytochemistry to examine the status of astrocytes and microglia in the cerebellum of Fmr1 mice during development and in adulthood.

**Results:**

We report increased expression of the astrocyte marker GFAP in the cerebellum of Fmr1 mice starting in the second postnatal week and persisting in to adulthood. At 2 weeks postnatal, expression of Tumor Necrosis Factor Receptor 2 (TNFR2) and Leukemia Inhibitory Factor (LIF) were elevated in the Fmr1 KO cerebellum. In adults, expression of TNFR2 and the glial marker S100*β* were also elevated in Fmr1 knockouts, but LIF expression was not different from wild‐type mice. We found no evidence of microglial activation or neuroinflammation at any age examined.

**Conclusions:**

These findings demonstrate an atypical pattern of astrogliosis in the absence of microglial activation in Fmr1 knockout mouse cerebellum. Enhanced TNFR2 and LIF expression in young mice suggests that changes in the expression of astrocytic proteins may be an attempt to compensate for delayed myelination in the developing cerebellum of Fmr1 mice.

## Introduction

The most abundant cells in the mammalian CNS, astrocytes serve a wide range of roles including contributing to the formation, maintenance, function and pruning of synapses, regulating neurotransmitter uptake, and maintaining the blood–brain barrier (reviewed in Molofsky et al. 2012; Pekny and Pekna 2014). CNS injury results in astrocyte activation, or reactive gliosis, which is characterized by hypertrophy of the astrocyte cell body and processes, and changes in gene expression, most notably upregulation of the intermediate filament Glial Fibrillary Acidic Protein (GFAP). These changes in astrocyte structure and function attempt to restrict and repair the damaged tissue (Pekny et al. 2014). Despite the growing list of important functions of astrocytes, the potential contributions of these cells to the pathology of diseases that have traditionally been considered “neuron diseases” have only recently begun to be explored.

Fragile X Syndrome, an X‐linked neurodevelopmental disorder, and the most common single gene cause of autism, results from loss of the RNA‐binding protein Fragile X Mental Retardation Protein (FMRP) (Hagerman et al. 2010; Hampson et al. 2012). FMRP is highly expressed in neurons throughout the CNS, where it binds to and regulates the translation, transport and stability of a subset of mRNAs (Bagni and Oostra 2013; Sethna et al. 2014). Until recently, research into the function of FMRP, and the consequences of its absence, have focused mainly on neurons. However, compounding evidence suggests an important role for FMRP in neural precursors (Callan et al. 2012) and glial cells (Pacey and Doering 2007; Gholizadeh et al. 2015), and that dysfunction of non‐neuronal cells of the CNS may be a key contributing factor in the pathogenesis of Fragile X Syndrome.

Oligodendrocyte Precursor Cells (OPCs) and mature oligodendrocytes express FMRP, albeit at lower levels than in mature neurons (Wang et al. 2004; Giampetruzzi et al. 2013; Pacey et al. 2013). Mice lacking FMRP show a 40% reduction in the number of OPCs in the cerebellum at postnatal day (PND) 15 and exhibit delayed myelination during development, which normalizes by 1 month of age (Pacey et al. 2013).

In astrocytes, FMRP is expressed during development and its expression is downregulated as the brain matures (Pacey and Doering 2007; Gholizadeh et al. 2015). Jacobs and Doering (2010) demonstrated that loss of FMRP in astrocytes leads to abnormal dendritic morphology and synapse development. Astrocyte dysfunction in the absence of FMRP may also contribute to the enhanced excitability of neurons in Fragile X syndrome. Yang et al. (2012) identified neurotrophin 3 (NT‐3) as a possible mediator of this effect. Release of NT‐3 is elevated in astrocytes from Fmr1 knockout (KO) mice, resulting in abnormal dendritic morphology and synaptic protein expression when these cells are cocultured with WT neurons. Loss of FMRP from astrocytes (but not neurons) leads to reduced expression of the glutamate transporter GLT‐1 (EAAT2) and subsequently reduced glutamate uptake by these cells (Higashimori et al. 2013), which could also contribute to neuronal hyperactivity and excitoxicity. Taken together, these studies demonstrate how astrocytes can influence neuronal structure and function in the absence of FMRP.

The majority of studies examining the consequences of loss of FMRP on astrocytes have been carried out in vitro. The objective of this in vivo study was to examine the status of astrocytes in the brains of Fmr1 KO mice during CNS development and in adulthood. The expression of astrocyte marker proteins in the cerebellum of Fmr1 KO mice were compared with wild‐type (WT) mouse cerebellum using quantitative western blotting and immunohistochemistry. In light of recent studies suggesting alterations in immune function in persons with Fragile X Syndrome (Ashwood et al. 2010; Careaga et al. 2014), we also assessed the status of microglia. Our results indicate an atypical pattern of glial alterations where Fmr1 mice display chronic, persistent activation of astrocytes with little or no activation of microglia. Fmr1 KO astrocytes also show upregulation of several proteins that promote myelination, suggesting that astrocyte activation may act, at least partially, to compensate for decreased myelination observed in Fmr1 KO cerebellum during early postnatal development.

## Materials and Methods

### Animals

All animal experiments were carried out in accordance with the guidelines set out by the Canadian Council on Animal Care and were approved by the University of Toronto Animal Care Committee. Wild‐type C57BL/6 and Fmr1 KO mice (backcrossed >10 generations on the C57BL/6 background) were generously provided by Dr. William Greenough, University of Illinois, and bred at the University of Toronto. All mice were the off‐spring of homozygous pairings.

### Immunohistochemistry

Postnatal day (PND) 15 or 30 or adult (2–4 months) WT and Fmr1 KO mice were anaesthetized with ketamine/xylazine and intracardially perfused with 0.1 mol/L PBS followed by 4% paraformaldehyde. Brains were removed and postfixed overnight in 4% paraformaldehyde at 4°C. For PND 7, pups were decapitated, and the brain/skull were fixed in 4% paraformaldehyde at room temperature for 5 h after which the brain was removed from the skull and further incubated in 4% paraformaldehyde at 4°C for approximately 20 h. Brains were subsequently rinsed with PBS and sunk in 30% sucrose/PBS overnight at 4°C. The cerebellum was removed, embedded in OCT, and sectioned with a cryostat. Floating coronal cerebellar sections (25 *μ*m) were rinsed in PBS then blocked for 1 h at room temperature in PBS containing 5% goat serum and 0.2% triton X‐100. After three 5 min washes, sections were incubated in primary antibody overnight at 4°C. Sections were washed in PBS then incubated in secondary antibody for 2 h at room temperature. After washing, sections were mounted on glass slides with Prolong Gold Antifade (Invitrogen, Waltham, MA). For PND 7, 25 *μ*m cryostat sections were thaw mounted on Superfrost slides (VWR) and treated as above.

All primary and secondary antibodies were diluted in PBS containing 5% goat serum (Sigma, St. Louis, MO). Primary antibodies included mouse anti‐Glial Fibrillary Acidic Protein (GFAP; clone N206A/8, 1:500; NeuroMab, Davis, CA); mouse anti‐S100*β* (1:10,000; Sigma); rabbit anti‐Iba1 (1:1500; Wako, Richmond, VA); rabbit anti‐TNFR2 (1:200; Acris, Hereford, Germany); rabbit anti‐LIF (1:200; Novus Biologicals, Littleton, CO) rabbit anti‐iNOS (1:2000; ThermoFisher Scientific, Waltham, MA); goat anti‐nNOS (1:1000; Novus Biologicals, Littleton, CO). Secondary antibodies were goat anti‐mouse Alexa‐Fluor 488 or 549 (1:2000); goat anti‐rabbit Alexa‐Fluor 488 or 549 (1:1000–1:2000); anti‐rat Dylight 488 (1:500); anti‐goat Alexa‐Fluor 488 (1:1000).

For quantitation of GFAP and S100*β* in the cerebellar cortex, digital images of the area of interest (simple lobule or Crus I) were captured using a Hamamatsu ORCA 285 CCD camera mounted on a Nikon E1000 microscope (Nikon Canada, Mississagua, Ontario, Canada) at 10× or 20× magnification with identical exposure times for all sections within each experiment. For quantitation of TNFR2 and GFAP in the cerebellar deep white matter, digital images were captured using a Nikon A1R Si Point Scanning Confocal microscope at 40× magnification, with identical acquisition settings for all sections within each experiment. For each experiment, animals were age and sex‐matched and matching sections from one WT and one Fmr1 KO brain were stained and imaged simultaneously. In each experiment, a total of 6–18 images per mouse were captured from 3 to 6 sections of each brain. Staining intensities were analyzed using Image J software (NIH, Bethesda, MD). For each image, a region of interest was drawn and the Mean Gray Value (sum of gray values divided by total number of pixels) was measured. For each pair of mice, the Mean Gray Values for all images were averaged for each genotype and expressed as a percentage of the WT value. The relative expression in KO mice is indicated as mean % WT expression ± SEM. A paired student's *T*‐test was used to determine statistical significance.

For colocalization of TNFR2 and GFAP the Thresholded Manders Coefficient (tM), which calculates the proportion of signal from one channel that overlaps with signal from the second channel, was determined using the Coloc2 plug in for Image J. For each pair, the average of the tM values for each genotype was calculated and expressed as the percent of the WT value. The mean ± SEM of these relative tM is presented as “percent colocalization”.

### Western blotting

Quantitative western blotting was performed as previously described (Adusei et al. 2010). Briefly, PND 7, 15, 30, or adult (2–4 month old) WT and Fmr1 KO were killed by cervical dislocation and the brains were removed and placed on ice. The cerebellum was isolated and homogenized in ice‐cold 50 mmol/L Tris‐HCl, 1% SDS, pH 7.4 supplemented with protease inhibitor cocktail (Roche, Lavel, Quebec, Canada) using a glass/teflon homogenizer. The protein concentration was determined using the BCA assay (Sigma). Equal amounts of protein (6–30 *μ*g depending on the abundance of the target protein) were loaded onto a 6, 10, or 12% polyacrylamide–SDS gel and transferred onto a nitrocellulose membrane after electrophoresis. The membranes were blocked in 5% milk for 1 h and incubated at 4°C overnight with one of the following primary antibodies: rabbit anti‐Iba1 (1:500; Wako) or rat anti‐CD68 (1:500; Serotec, Oxford, U.K.) and mouse anti‐GAPDH antibody (1:40,000–1:100,000; Sigma). After washing, a goat anti‐mouse, goat anti‐rabbit or goat anti‐rat (Jackson Labs, Bar Harbor, ME) HRP‐conjugated secondary antibody was applied for 2 h. The immunoreactive proteins were visualized using the FluorChem™ MultiImage Light Cabinet (Alpha Innotech). Densitometric analysis was carried out using the AlphaEaseFC software (Alpha Innotech, San Jose, CA). The intensity of the band of interest was normalized relative to the GAPDH band intensity. Protein expression in WT and Fmr1 KO animals is presented as a percentage of WT expression levels. An unpaired Student's *t‐*test was performed to determine statistical significance.

### LPS immune challenge

Adult, female WT and Fmr1 mice were administered a single intraperitoneal injection of 1 mg/kg lipopolysaccharide (LPS; Sigma) or sterile saline in a volume of 0.1 mL/10 g body weight. Seventy‐two hours after the injection, the mice were intracardially perfused and immunohistochemistry was performed on cerebellar sections using an anti‐Iba1 antibody as described above. For each experiment, one animal was examined from each experimental group (WT saline, Fmr1 saline, WT LPS, and Fmr1 LPS) simultaneously and the results from each condition are expressed as a percent of WT saline.

## Results

### Astrocyte markers are elevated in Fmr1 mouse cerebellum

**Figure 1:**
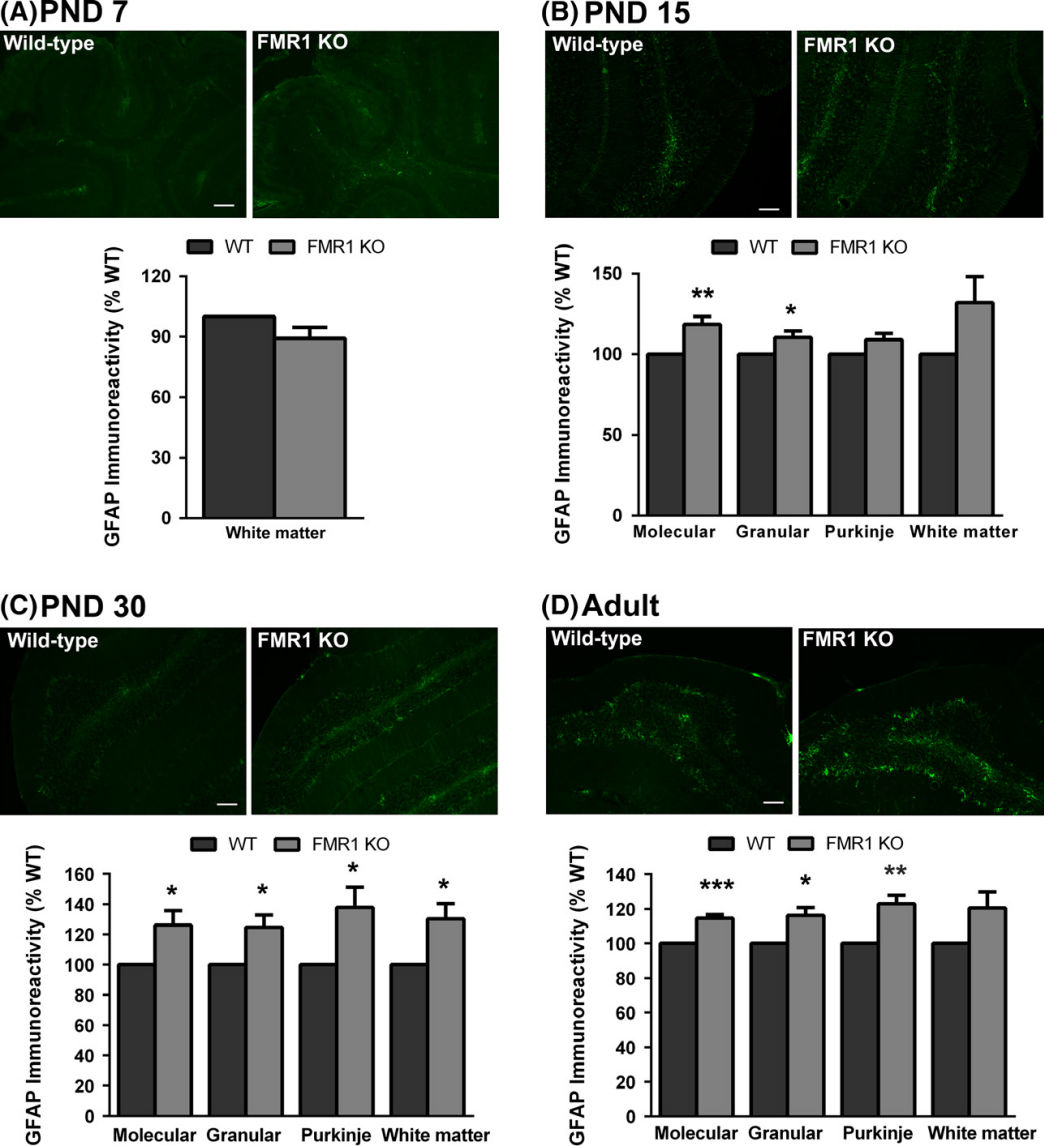
GFAP expression is elevated in Fmr1 cerebellum. Immunocytochemical analysis of the astrocyte marker GFAP was examined in the cerebellum of wild‐type and Fmr1 KO mice at PND 7, 15, 30, and adult. There was no significant difference in GFAP expression at PND 7 (A), whereas GFAP expression was significantly increased in the cerebellar cortex of Fmr1 mice at PND 15 (B), PND 30 (C), and adult (D). Representative cerebellar sections shown are from an age‐ and sex‐matched pair of mice at each age. Scale bar = 100 *μ*m. *N* = 4–7 per sex per genotype. *P < 0.05; **P < 0.01; ***P < 0.001.

To study the effect of loss of FMRP on astrocytes, we examined the expression of the astrocyte‐specific marker GFAP in the cerebellum of WT and Fmr1 mice at PND 7, 15, 30, and adult by immunohistochemistry. GFAP immunoreactivity was quantified in two regions of the posterior cerebellar cortex—the simple lobule and Crus I—as well as the white matter within these lobules. The data presented below is for the simple lobule. Overall, the Crus I showed similar trends of GFAP expression (data not shown). In the PND 7 cerebellum, GFAP immunoreactivity was low relative to other time points and was primarily restricted to the white matter tracts; therefore only this region was quantified. GFAP expression did not differ between WT and Fmr1 at PND 7 (Fig. 1A; 89 ± 5.4% of WT, *P* = 0.12). By PND 15, GFAP expression was significantly elevated in the molecular (Fig. 1B; 119 ± 4.9%, *P* = 0.009) and granule cell (110 ± 4.0%, *P* = 0.04) layers. A trend toward an increase in GFAP expression was also seen in the Purkinje cell layer (109 ± 3.8%, *P* = 0.06) and white matter tracts (132 ± 16.2%, *P* = 0.10) at PND 15.

At PND 30, compared to WT mice, Fmr1 mice showed a significant elevation in GFAP expression in all three layers of the cerebellar cortex (Fig. 1C; molecular: 126 ± 9.5%, *P* = 0.03; granular: 125 ± 8.2%, *P* = 0.02; Purkinje: 138 ± 13.4%, *P* = 0.02), and in the white matter (130 ± 10.2, *P* = 0.02). In the adult (2–4 months old), there was a significant increase in GFAP expression in all three layers of the cerebellar cortex in Fmr1 mice (Fig. 1D; molecular: 115 ± 2.1%, *P* = 0.00025; granular: 116 ± 4.6%, *P* = 0.03; Purkinje: 123 ± 4.8%, *P* = 0.006) and a trend toward an increase in the white matter (121 ± 9.2%, *P* = 0.06). Together, these data demonstrate an increase in GFAP expression in the cerebellum of Fmr1 mice beginning in the second week postnatal.

**Figure 2:**
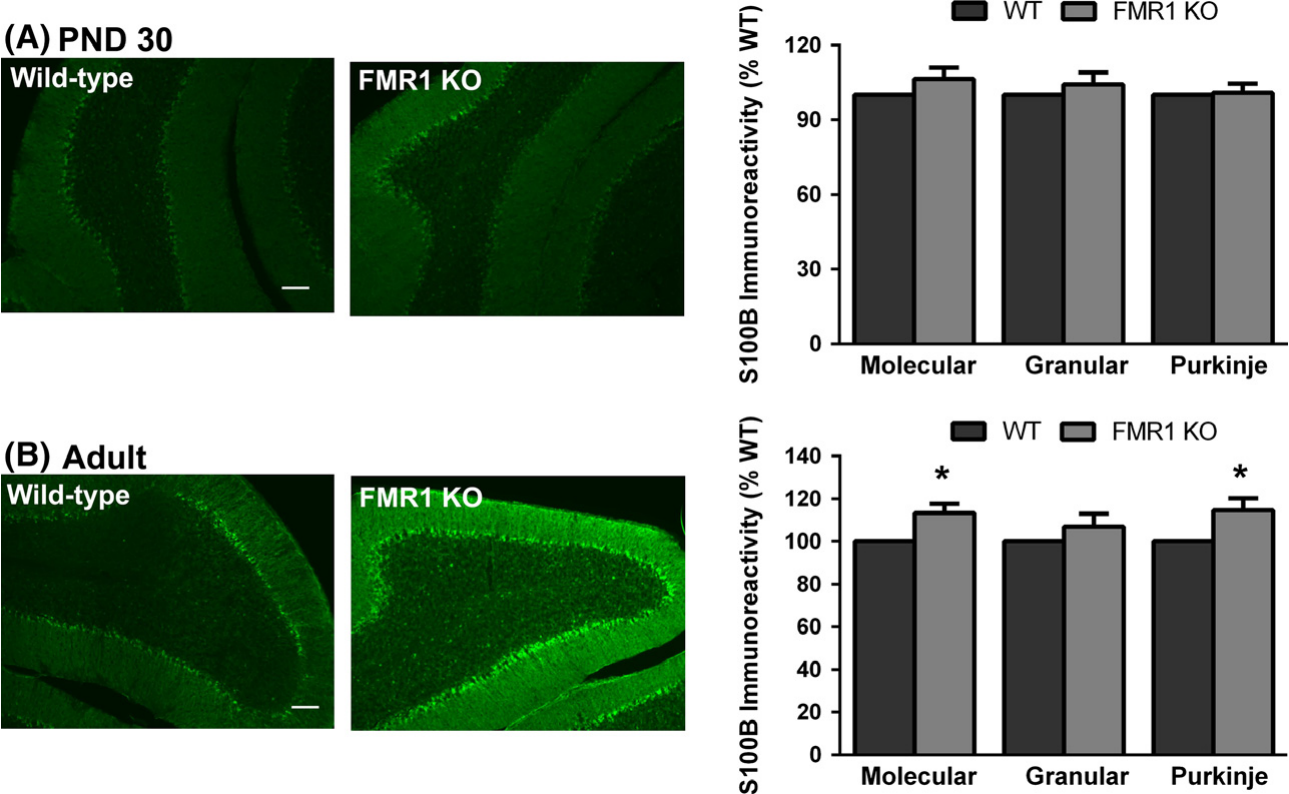
S100*β* expression is increased in Fmr1 cerebellum. Immunocytochemical staining for S100*β*, a glial marker that labels primarily Bergmann glia cell bodies in the cerebellum, was examined at PND 30 and in adult mice. No significant differences in expression of S100*β* were detected at PND 30 (A), but S100*β* expression was increased in the molecular and Purkinje cell layers of the adult KO mouse compared to WT (B). Scale bar = 100 *μ*m. *N* = 4–8 per genotype. *P < 0.05.

To further examine the effect of the absence of FMRP on glial cells, we immunostained cryostat sections of WT and Fmr1 cerebellum with an antibody to the calcium‐binding protein and glial‐selective marker S100*β* at different developmental time points. In the cerebellum, S100*β* predominantly labels Bergmann glia. We have previously shown that Bergmann glia do not express FMRP (Pacey et al. 2013). Labeling for S100*β* was not detectable at PND 7. At PND 30, S100*β* expression was easily detectable in the mouse cerebellar cortex, but the levels were not different between WT and Fmr1 in any of the three layers (Fig. 2A; molecular layer: 106 ± 4.5%, *P* = 0.25; granule cell layer: 104 ± 4.8%, *P* = 0.44; Purkinje layer: 101 ± 3.7%, *P* = 0.82). However, there was a significant increase over WT in S100*β* expression in the adult Fmr1 molecular (113 ± 6.1%, *P* = 0.03) and Purkinje cell layers (Fig. 2B; 115 ± 7.9%, *P* = 0.04), which correspond to the regions containing the processes and cell bodies of Bergmann glia, respectively. There was no significant difference in S100*β* expression in the granular layer of adult Fmr1 mice compared to WT mice (107 ± 8.6, *P* = 0.31). Taken together, these findings demonstrate overexpression of both GFAP and S100*β* in astrocytes and Bergmann glia in Fmr1 mice.

### Microglia are not changed in the Fmr1 cerebellum

**Figure 3:**
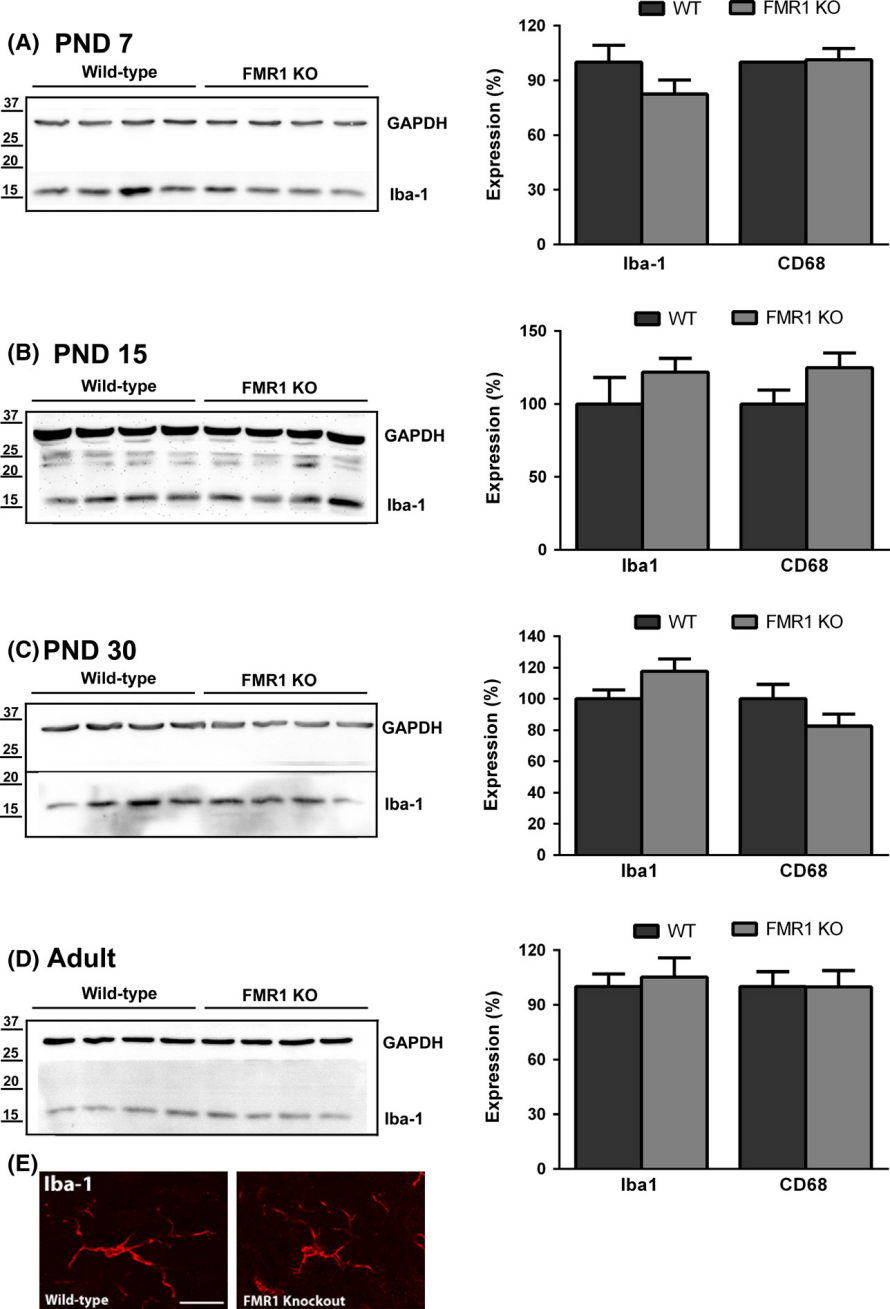
No changes in microglia in Fmr1 cerebellum. The expression of the microglial markers CD68 and Iba‐1 was assessed in the cerebellum by immunohistochemistry and western blot. No significant differences in CD68 or Iba‐1 expression were detected at PND 7 (A), PND15 (B), PND30 (C), or adults (D), and no obvious morphological differences were evident in Iba‐1 positive cells from WT or Fmr1 at any time point examined (E; representative image from PND 30). Scale bar = 20 *μ*m. *N* = 6–12 per genotype.

Classic models of neuroinflammation involve an initial activation of microglia as a prelude to astrocyte activation. To assess the effects of the loss of FMRP on microglia, the expression of two microglial markers, CD68 and Iba‐1, was examined in the cerebellum of WT and Fmr1 mice at each developmental time point using quantitative western blotting (Fig. 3A–D). There were no significant differences in Iba‐1 or CD68 expression at any time point examined. Microglia undergo a dramatic change in morphology from a ramified resting phenotype to an amoeboid structure when activated. On cryostat sections of the cerebellum, labeling with the anti‐Iba‐1 antibody revealed no detectable differences in microglial morphology in the Fmr1 cerebellum compared to WT (Fig. 3E).

To determine if microglia from Fmr1 mice respond differently to an immunological challenge, we injected adult WT and Fmr1 mice i.p. with 1 mg/kg lipopolysaccharide (LPS) and examined the brains for Iba‐1 expression 72 h after stimulation. Consistent with western blot results, the level of Iba‐1 staining in the deep cerebellar white matter of saline‐injected WT and Fmr1 mice was not different (Fig. S1, Fmr1 = 98 ± 0.85% of WT), and no differences in microglial morphology were observed between the genotypes. LPS injection induced a large increase in Iba‐1 expression in both WT (LPS = 133 ± 0.32% of WT saline) and Fmr1 mice (LPS = 128 ± 16.5% of Fmr1 saline); however, the magnitude of this increase was not different between the genotypes indicating that microglia from Fmr1 mice responded similar to WT microglia under these conditions. Taken together, these results indicate that no major alterations of microglia are apparent in the cerebellum of Fmr1 mice.

### No induction of NOS in Fmr1 KO astrocytes

Since classic astrocyte activation is known to result in increased production of nitric oxide through induction of inducible nitric oxide synthase (iNOS) (Luth et al. 2001) and possibly neuronal NOS (nNOS) (Catania et al. 2003; Carbone et al. 2008), we examined the expression of these two proteins in the cerebellar deep white matter of PND 15 and adult WT and Fmr1 mouse. At both ages and in both genotypes, the expression of iNOS in white matter astrocytes was barely detectable and below the level that would allow for accurate quantification (data not shown). While nNOS was highly expressed in cerebellar neurons, no expression was detected in astrocytes in the deep white matter of WT or Fmr1 mice at any age examined. If astrocytes in Fmr1 KO mice were undergoing a classic functional activation, we would expect to see a large increase in NOS expression (Bal‐Price and Brown 2001) compared to WT. The absence of any obvious NOS induction again suggests an atypical pattern of astrocyte activation in the cerebellum of Fmr1 KO mice, which does not involve increased nitric oxide production.

### Tumor necrosis factor receptor 2 and leukemia inhibitory factor are elevated in Fmr1 mouse brain

**Figure 4:**
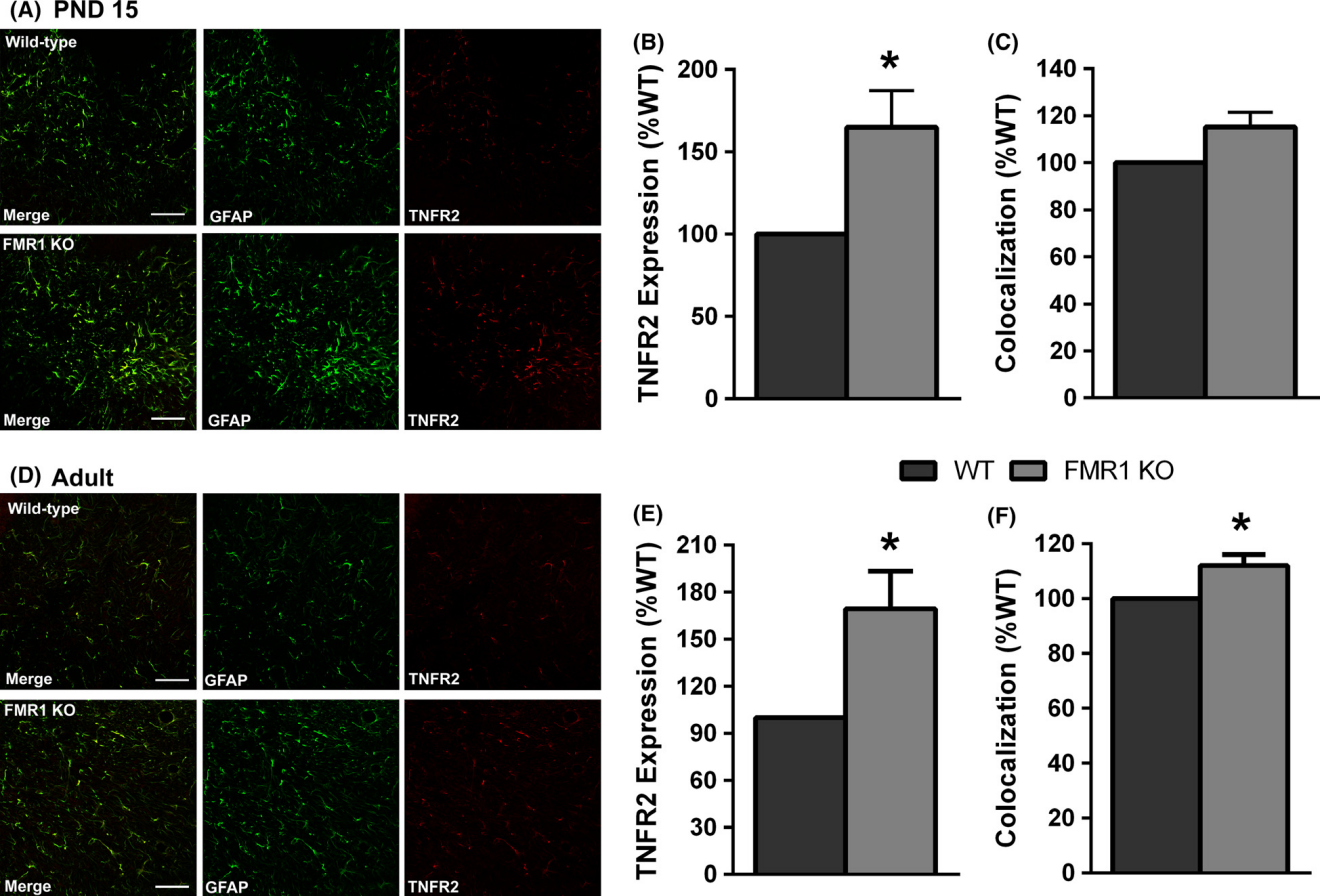
TNFR2 expression is elevated in Fmr1 KO astrocytes. TNFR2 expression was assessed in the deep white matter of the cerebellum using immunohistochemistry. (A, D) Representative images of TNFR2 and GFAP expression in the cerebellar deep white matter of wild‐type and Fmr1 KO mice at PND 15 (A) and adult (D). Expression of TNFR2 was significantly elevated in the Fmr1 KO at PND 15 (B) and adult (E). Colocalization of TNFR2 and GFAP was also increased in Fmr1 KO cerebellum at PND 15 (C) and adult (F), indicating increased TNFR2 expression by KO astrocytes. Scale bar = 50 *μ*m. *N* = 5 per genotype. *P < 0.05.

Tumor necrosis factor alpha (TNF*α*) is a cytokine released by astrocytes and microglia during neuroinflammation. The cellular effects of TNF*α* are mediated through two receptors—TNFR1 and tumor necrosis factor receptor 2 (TNFR2). TNFR1 is thought to mediate the proinflammatory effects of TNF, whereas TNFR2 mediates the anti‐inflammatory and promyelinating effects (Naude et al. 2011; Wang and Al‐Lamki 2013). TNFR2 is expressed by astrocytes where its activation has been shown to stimulate the release of Leukemia Inhibitory Factor (LIF) which promotes the maturation of OPCs leading to increased myelination (Fischer et al. 2014). Since myelination is delayed in the cerebellum of Fmr1 mice (Pacey et al. 2013) we postulated that, in the absence of FMRP, astrocytes might upregulate the expression of TNFR2 to compensate for reduced myelination seen early in postnatal development. To examine this, we immunostained cryostat sections from PND 15 and adult WT and Fmr1 mouse cerebellum with antibodies to TNFR2 and GFAP and examined the expression. TNFR2 was most highly expressed in the deep cerebellar white matter, a region that also contains fibrous astrocytes in close proximity to oligodendrocytes and OPCs, so we focused our analysis on this region. TNFR2 expression was robustly increased in the deep cerebellar white matter of Fmr1 mice at both PND 15 (Fig. 4C; 165 ± 22.3% of WT, *P* = 0.04) and in adults (Fig. 4G; 165 ± 22.4% of WT, *P* = 0.04). GFAP expression was also increased in the deep white matter at PND 15 (145 ± 13.3% of WT, *P* = 0.03), but this did not quite reach significance in the adult (141 ± 21.4% of WT, *P* = 0.13). We then examined the colocalization of GFAP and TNFR2 and found an increase in the proportion of GFAP positive cells/processes that also expressed TNFR2 in the deep white matter of both PND 15 (Fig. 4D; 115 ± 6.3% of WT, *P* = 0.07) and adult (Fig. 4H; 112 ± 3.9% of WT, *P* = 0.04) Fmr1 KO cerebellum. Together, these data demonstrate an increase in the expression of TNFR2 in astrocytes in the young and adult Fmr1 cerebellum.

**Figure 5:**
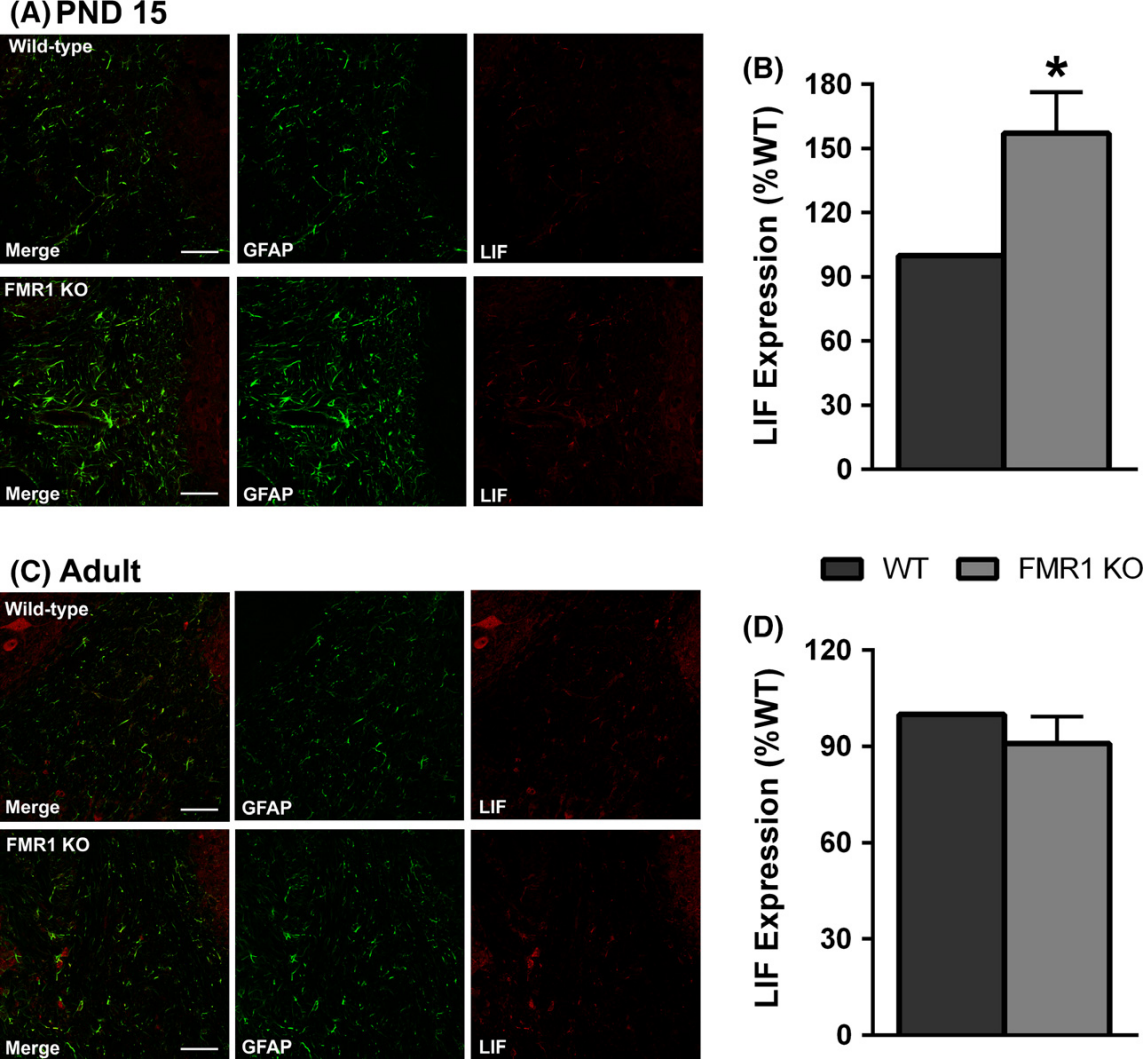
LIF expression is increased in Fmr1 KO astrocytes during development but not in adulthood. LIF expression was measured in the deep white matter of the cerebellum using immunohistochemistry. (A, C) Representative images of LIF and GFAP expression in the cerebellar deep white matter of wild‐type and Fmr1 KO mice at PND 15 (A) and adult (C). LIF expression was significantly elevated in the Fmr1 KO at PND 15 (B) but was not different from WT controls in adult (D). Scale bar = 50 *μ*m. *N* = 5 per genotype. *P < 0.05.

We then examined the expression of LIF in the cerebellar deep white matter of WT and Fmr1 mice. LIF was highly expressed in the molecular layer of the cerebellar cortex with much lower expression in the cerebellar white matter. However, given that the increase in TNFR2 expression was largely confined to the white matter, LIF expression was quantified only in this region. The majority of LIF in the deep cerebellar white matter colocalized with GFAP, indicating much of the LIF expression was present in astrocytes. However, the intensity of the LIF signal was not strong enough relative to GFAP to allow for an accurate measurement of colocalization using Image J software. At PND 15, LIF expression was significantly increased in Fmr1 KO mice (Fig. 5C; 157 ± 19.2%, *P* = 0.04). However, there was no difference in LIF expression between the genotypes in adult mice (Fig. 5F; 91 ± 4.2%, *P* = 0.12). Together, these findings are consistent with a model where astrocytes respond to reduced myelination in the Fmr1 cerebellum by upregulating TNFR2 expression, resulting in increased LIF secretion, OPC maturation, and enhanced myelination.

## Discussion

In the mouse brain, FMRP is expressed in astrocytes during development, with expression being downregulated as the brain matures (Pacey and Doering 2007; Gholizadeh et al. 2015). In vitro, loss of FMRP in astrocytes can negatively affect the structure and function of neurons (Jacobs and Doering 2010; Yang et al. 2012; Higashimori et al. 2013); however, the effects of the loss of FMRP on astrocytes in vivo have not been extensively studied. Here, we report the activation of astrocytes in the cerebellum of Fmr1 KO mice beginning at 2 weeks postnatal and persisting into adulthood. Although the current study focused on an analysis of the cerebellum, our preliminary results indicate that the elevated markers for astrocytes extend to other brain regions (e.g., hippocampus, cortex) in the Fmr1 CNS (data not shown).

The issue of whether mRNAs for GFAP and TNFR2 interact with FMRP was not addressed in our study, although two other studies that did examine FMRP mRNA substrates did not list either mRNA (Darnell et al. 2011; Ascano et al. 2012). Although FMRP is expressed in astrocytes early in development, by adulthood FMRP levels in astrocytes are, with the exception of the corpus callosum, negligible in most brain regions including the cerebellum (Pacey and Doering 2007; Gholizadeh et al. 2015). Therefore, while we cannot rule out an effect of FMRP on translation of glial mRNAs, it is unlikely to be the exclusive cause of elevated astrocyte protein expression in the adult brain. Although the elevation in GFAP (and S100*β*) may not be a consequence of direct regulation of astrocyte markers by FMRP, it might instead be induced indirectly. One possibility to consider in future studies is dysregulated intracellular calcium. GFAP expression rises in response to increased intracellular calcium (Lee et al. 2000). Tessier and Broadie (2011) have shown aberrant intracellular calcium homeostasis in drosophila neurons lacking FMRP, and abnormally enhanced asynchronous calcium oscillations have been demonstrated in astrocytes prepared from mice with the Fmr1 premutation gene expansion (i.e., fragile X‐associated tremor and ataxia syndrome; Cao et al. 2013).

Upregulation of GFAP expression is a salient characteristic of astrocyte activation and typically occurs in response to CNS injury. Astrocyte activation is usually accompanied by activation of microglia and the production of several pro‐ and anti‐inflammatory factors, including cytokines, growth factors, and nitric oxide (Bal‐Price and Brown 2001; Pekny et al. 2014). Signs of neuroinflammation (e.g., activated astrocytes and microglia) have been reported in postmortem studies of the brains of autistic individuals (Vargas et al. 2005; Li et al. 2009; Ashwood et al. 2011) and evidence of peripheral immune activation has also been demonstrated in individuals with idiopathic autism (reviewed in Goines and Van de Water 2010; Onore et al. 2012). However, our results in the fragile X mouse brain show that activation of astrocytes in the cerebellum occurs independent of changes in microglia. We also saw no evidence of increased production of inflammatory cytokines in the plasma or cerebellar homogenates from Fmr1 mice (data not shown) and Fmr1 astrocytes did not show elevated nitric oxide synthase type 1 (“nNOS”) or type 2 (“iNOS”) both of which typically show considerable induction in activated astrocytes (Bal‐Price and Brown 2001; Catania et al. 2003; Carbone et al. 2008). These findings are at odds with traditional neuroinflammation and suggest a pattern of atypical astrocyte activation in the cerebellum of Fmr1 mice.

Our observations are consistent with, and extend those of Yuskaitis et al. (2010) who reported GFAP activation in the adult Fmr1 mouse forebrain but no changes in cultured microglia or peripheral immune activation. Also of note are the results from a study on postmortem brain samples from subjects with idiopathic autism where in the prefrontal cortex markers for both astrocytes and microglia were increased, whereas in the cerebellum only astrocyte markers were elevated (Edmonson et al. 2014). Forbes‐Lorman et al. (2014) showed that transient knockdown of MeCP2 (the protein implicated in another syndromic form of autism, Rett Syndrome) led to specific changes in astrocyte protein expression which included upregulation of GFAP but no changes in S100*β* or vimentin expression. It is therefore possible that loss of FMRP (either directly from astrocytes or secondary to loss in neurons) leads to upregulation of a subset of genes/proteins in astrocytes (including GFAP, S100*β*, TNFR2 and LIF) that is distinct from the classic neuroinflammatory response associated with CNS injury or immune stimulation. Nevertheless, it is conceivable that, while different processes may be at play in Fragile X Syndrome versus idiopathic autism, shared alterations in astrocyte function may be important in eliciting some of the phenotypic commonalities between the two disorders.

In the cerebellum, Fmr1 mice show delayed myelination during development, which normalizes after the first postnatal month (Pacey et al. 2013). Astrocytes are important for the promotion and maintenance of myelination. Liedtke et al. (1996) reported abnormal white matter architecture, aberrant myelination, and late‐onset myelin loss in GFAP null mice. In the Fmr1 cerebellum, overexpression of GFAP first becomes evident in the second postnatal week, which also correlates with reduced myelin levels in Fmr1 cerebellum. Given the importance of GFAP for myelination, we speculate that reduced myelination during cerebellar development could be the stimulus for GFAP overexpression and astrocyte activation in Fmr1 mice. The persistence of GFAP upregulation into adulthood might reflect the fact that GFAP is required for proper maintenance of myelin (Liedtke et al. 1996).

Liedtke et al. (1996) suggested that GFAP regulates the secretion of a specific factor or factors from astrocytes that is important for myelination. Ishibashi et al. (2006) later showed that astrocytes could promote myelination by releasing LIF in response to ATP generated by neuronal activity. They speculated that LIF might be the “unknown” secreted factor regulated by GFAP. Fischer et al. (2011) demonstrated that astrocytes could also promote myelination by secreting LIF in response to activation of TNFR2 through a PI3K/Akt‐dependent mechanism. We observed increased LIF expression in the cerebellum of Fmr1 mice at PND 15, an age at which we have previously shown reduced myelination in these mice (Pacey et al. 2013). In contrast, LIF levels were normal in adults, where there is no longer a myelin deficit in the cerebellum. TNFR2 expression was also elevated at PND 15, and could therefore act as the stimulus for LIF overproduction at this age. Elevated LIF expression could also result from other factors, including elevated PI3K activity. Increased PI3K activity has been demonstrated in neurons and non‐neuronal cells in the absence of FMRP (Gross et al. 2010). Elevated PI3K activity associated with upregulation of metalloproteinase 9, induces increased activity of AKT and mTOR which in turn impairs dendritic spine maturation in Fmr1 mice (Gkogkas et al. 2014; Sidhu et al. 2014). Elevated PI3K activity in astrocytes could drive the increased LIF expression alone, or in combination with the elevated TNFR2 activation. Taken together, these findings suggest that changes in astrocytes—specifically upregulation of GFAP, TNFR2 and/or LIF expression—could be a compensatory mechanism to off‐set the reduced myelination present in Fmr1 cerebellum in the first few postnatal weeks.

Although we postulate a positive role for astrocyte activation on myelination in Fmr1 cerebellum, it is also possible that changes in astrocyte function could negatively contribute to the neuronal pathology characteristic of Fragile X Syndrome. In vitro, neurons show morphological alterations, functional deficits, and abnormal synapse development when cocultured with astrocytes from Fmr1 KO mice (Jacobs and Doering 2010; Jacobs et al. 2010; Yang et al. 2012; Higashimori et al. 2013). Coculturing neurons lacking FMRP with astrocytes from WT mice can rescue some of the morphological abnormalities associates with Fragile X neurons (Jacobs and Doering 2010), suggesting that at least some of the alterations associated with Fragile X astrocytes are detrimental to neurons. Astrocytes also express several proteins that have been implicated in the neuropathology of Fragile X and related disorders, including matrix metalloproteinase 9 (MMP9; Noble et al. 2002) and amyloid precursor protein (Siman et al. 1989). Reducing the levels of either MMP9 (Bilousova et al. 2009), or amyloid precursor protein (Westmark et al. 2011) in Fmr1 mice has been shown to alleviate several behavioral (e.g., audiogenic seizures, anxiety) and morphological (immature dendritic spines) abnormalities associated with Fragile X Syndrome, suggesting the abnormal production of certain proteins by astrocytes could be pathogenic in Fragile X.

We report an atypical pattern of astrocyte activation in the cerebellum of Fmr1 mice, highlighted by chronic overexpression of GFAP, and not accompanied by activation of microglia in the cerebellum of Fmr1 mice. Upregulation of proteins known to promote myelination at developmental time points where Fmr1 mice show reduced myelination, suggests that astrocyte activation might provide a mechanism to compensate for reduced myelin levels during early postnatal development. Although FMRP is expressed in many astrocytes throughout the developing mouse brain, astrocytic expression is restricted to a few brain regions, such as the corpus callosum, in the adult CNS (Gholizadeh et al. 2015). It is not clear whether the changes in astrocytes observed in this study are a direct consequence of the loss of FMRP in astrocytes, or result indirectly from the loss in neurons—or a combination of both. Further in vivo studies examining mutant mice with specific deletions of FMRP in neurons versus astrocytes are needed to more fully elucidate the causes and consequences of astrogliosis in Fmr1 cerebellum. Nevertheless, these findings further validate astrocytes as important players in Fragile X Syndrome and identify these cells as possible targets for future therapies.

## Conflict of Interest

None declared.

## Supporting information


**Figure S1.** Immune challenge in WT and Fmr1 KO mouse cerebellum.
