# Supplementary figures and images for: Persistent astrocyte activation in the fragile X mouse cerebellum
Source: Brain Behav. 2015 Sep 25;5(10):e00400. doi: 10.1002/brb3.400 (PMC4614053; doi:10.1002/brb3.400)

**Supplementary figure 1**

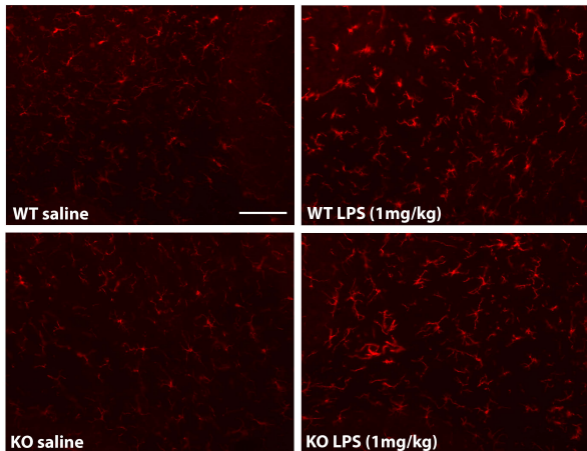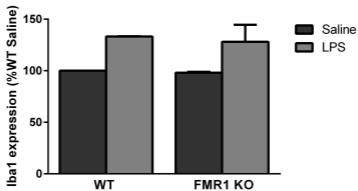

Supplement: Supplementary file 1 — Figure S1. Immune challenge in WT and Fmr1 KO mouse cerebellum. [file BRB3-5-0b-s001.pdf]
